# Investigating the Associations Between COVID-19, Long COVID, and Sleep Disturbances: Cross-Sectional Study

**DOI:** 10.2196/53522

**Published:** 2024-12-13

**Authors:** Heng Shao, Hui Chen, Kewang Xu, Quan Gan, Meiling Chen, Yanyu Zhao, Shun Yu, Yutong Kelly Li, Lihua Chen, Bibo Cai

**Affiliations:** 1 Department of Geriatrics, The First People’s Hospital of Yunnan Province, Kunming, China; 2 The Affiliated Hospital of Kunming University of Science and Technology, Kunming, China; 3 Department of Clinical Psychology, The First People’s Hospital of Yunnan Province, Kunming, China; 4 Medical College, Kunming University of Science and Technology, Kunming, China; 5 Faculté de Médecine, Université Paris Saclay, Paris, France; 6 Rehabilitation Department of Chinese Medicine, Chengjiang People’s Hospital, Yuxi, China; 7 School of Mathematics and Statistics, Yunnan University, Kunming, China; 8 Department of Biochemistry and Psychology, Mount Holyoke College, South Hadley, MA, United States; 9 School of Medicine, Stanford University, Stanford, CA, United States; 10 Epidemic Surveillance and Public Health Emergency Response Center, Yunnan Center for Disease Control and Prevention, Kunming, Yunnan, China; 11 Department of Traditional Chinese Medicine, The First People’s Hospital of Yunnan Province, Kunming, China

**Keywords:** COVID-19, long COVID, sleep disturbances, psychological outcomes, socioeconomic factors, cross-sectional study

## Abstract

**Background:**

COVID-19 has not only resulted in acute health issues but also led to persistent symptoms known as long COVID, which have been linked to disruptions in sleep quality.

**Objective:**

This study aims to investigate the associations between COVID-19, long COVID, and sleep disturbances, focusing on demographic, socioeconomic, and psychological factors among a Chinese population.

**Methods:**

This cross-sectional study included 1062 participants from China. Demographic, socioeconomic, and clinical data were collected through web-based questionnaires. Participants were divided into 2 groups based on COVID-19 infection status: infected and noninfected. Within the infected group, participants were further categorized into those with long COVID and those without long COVID. Noninfected participants were included in the non–long COVID group for comparison. Sleep quality was assessed using the Pittsburgh Sleep Quality Index (PSQI), while depression and anxiety were evaluated using the Patient Health Questionnaire-9 (PHQ-9) and the Generalized Anxiety Disorder-7 (GAD-7) scales, respectively. Multivariable linear regression was conducted to examine the associations between COVID-19, long COVID, and sleep quality, adjusting for demographic and psychosocial factors.

**Results:**

COVID-19 infection was confirmed in 857 participants, with 273 of them developing long COVID. No significant sex disparities were observed in infection rates (*P*=.63). However, a marginal statistical difference was noted in the prevalence of long COVID among females (*P*=.051). Age was significantly associated with both infection rates (*P*<.001) and long COVID (*P*=.001). Participants aged 60‐70 years were particularly vulnerable to both outcomes. Sleep latency was significantly longer in the infected group (mean 1.73, SD 0.83) compared to the uninfected group (mean 1.57, SD 0.78; *P*=.01), and PSQI scores were higher (mean 8.52, SD 4.10 vs. 7.76, SD 4.31; *P*=.02). Long COVID participants had significantly worse sleep outcomes across all metrics (*P*<.001), except for sleep medication use (*P*=.17).

**Conclusions:**

Our findings indicate that long COVID is strongly associated with significant sleep disturbances, while initial COVID-19 infection shows a more moderate association with sleep issues. Long COVID–related sleep disturbances were exacerbated by factors such as age, income, and chronic health conditions. The study highlights the need for targeted interventions that address the multifaceted impacts of long COVID on sleep, especially among vulnerable groups such as older adults and those with lower socioeconomic status. Future research should use longitudinal designs to better establish the temporal relationships and causal pathways between COVID-19 and sleep disturbances.

## Introduction

As of September 2023, the World Health Organization has reported approximately 771 million confirmed COVID-19 cases worldwide, along with nearly 7 million deaths globally [1]. Following SARS-CoV-2 infection, a wide spectrum of clinical symptoms can arise due to viral replication and its impact on the respiratory system. These symptoms typically include fever, cough, and shortness of breath but can escalate to severe complications such as pneumonia, acute respiratory distress syndrome, and multi-organ failure [2]. After the acute infection phase, the recovery stage can range from fatigue and cardiovascular issues to more complex conditions like thrombosis, cerebrovascular diseases, autonomic nervous system dysfunction, and cognitive impairments [3,4]. The term “long COVID” entered the public lexicon in 2020 when Elisa Perego used it to describe her experience with persistent, cyclical, and multisystemic symptoms. This term rapidly gained traction on social media platforms [5]. Since then, scholarly discourse on the persistence of symptoms post–SARS-CoV-2 infection has intensified [6].

In response, the World Health Organization released the Delphi Consensus in 2021, which synthesized prevailing perspectives on “post-COVID syndrome” or “long COVID” [7]. Although the topic remains under debate, a prevailing consensus suggests that long COVID manifests in individuals previously infected with SARS-CoV-2, with symptoms typically appearing within 3 months postinfection. These manifestations persist beyond 2 months and cannot be attributed to other medical conditions. Commonly reported manifestations encompass fatigue, respiratory challenges, and cognitive disturbances, which significantly hinder routine daily functions. This consensus is considered useful to foster research while recognizing that “long COVID” may not be a single entity [8].

The global burden of disease during the COVID-19 pandemic has revealed a marked increase in psychological disorders such as depression and anxiety. By 2020, the global incidence of severe depression had risen by 27.6%, while anxiety cases increased by 25.6% [9]. Recent studies suggest that the onset of anxiety and depression post–COVID-19 infection is influenced by a combination of factors, including gender, stress levels, socioeconomic status, and other societal variables [10-12]. Furthermore, both pre-existing and pandemic-related anxiety and depression are not only potential consequences of COVID-19 but also significant predictors of developing long COVID [13]. The presentation and severity of depressive and anxiety symptoms vary considerably in the early stages of COVID-19 infection, with symptoms evolving as the disease progresses [14-16].

Insomnia is frequently identified as a prominent symptom, leading patients with mood disorders such as depression and anxiety to seek medical care [17]. A meta-analysis found that during the COVID-19 pandemic, 52.57% of the population experienced insomnia symptoms, with 16.66% meeting the criteria for clinically significant insomnia. Of these, 13.75% presented with moderate symptoms, and 2.5% with severe symptoms [18]. Another meta-analysis of 29 studies involving 13,935 patients with long COVID found that 46% experienced sleep disturbances, with poor sleep quality affecting 56%, insomnia 38%, and excessive daytime sleepiness 14% of participants [19]. Despite the growing body of research on the health impacts of COVID-19, there remains some gap in understanding the specific effects of long COVID on sleep quality, and how this relates to psychological factors such as depression and anxiety. Moreover, the interplay between demographic and socioeconomic factors and their contributions to sleep disturbances among patients with long COVID has not been fully explored. This study seeks to fill these gaps by conducting a comprehensive cross-sectional analysis of individuals affected by long COVID. Specifically, we aim to assess the prevalence and severity of sleep disturbances, examine the relationship between long COVID and psychological outcomes such as depression and anxiety, and identify key demographic and socioeconomic factors contributing to insomnia and poor sleep quality in this population.

## Methods

### Design and Data Collection

In China, the government officially ended its zero-COVID-19 policy on January 8, 2023 [20]. Following this, nucleic acid test results for SARS-CoV-2 showed an initial rise in positivity rates, followed by a gradual decline. Notably, from December 22 to 25, 2022, there was a surge in positive cases, which then fluctuated before eventually decreasing [21]. In response to these developments, we initiated a cross-sectional study in March 2023. This timing was strategically chosen to include individuals who may have contracted the virus for the first time after the policy change, as well as those who had managed to remain uninfected during the earliest waves. Additionally, participants previously infected by this point would likely have been experiencing symptoms for more than 4 weeks. This study followed the Strengthening the Reporting of Observational Studies in Epidemiology (STROBE) guidelines.

The survey instrument we developed covered a broad array of factors, including sex, ethnicity, age, income, education, location, occupation, chronic conditions (based on medical insurance records), history of mental health issues, vaccination status, date of COVID-19 infection, the need for hospitalization or ICU admission, medications taken during infection, and the persistence and nature of symptoms beyond 4 weeks postinfection [22]. To assess psychological well-being, we used several self-assessment tools: the Patient Health Questionnaire-9 (PHQ-9), the Generalized Anxiety Disorder-7 (GAD-7), and the Pittsburgh Sleep Quality Index (PSQI). Additionally, the Multidimensional Scale of Perceived Social Support (MSPSS) and the Alcohol Use Disorders Identification Test (AUDIT) were included in our assessment.

On March 7, 2023, we conducted a pilot test with 18 participants to validate the effectiveness of the scales and ensure their suitability before the official launch. Following this step, for the formal survey, we prioritized smartphone users by entering all survey information into the “Wenjuanxing” web-based platform [23]. Each question was set as mandatory, and incomplete forms could not be submitted. A QR code was generated and distributed via social media platforms, hospitals, communities, and shopping centers to encourage people from diverse backgrounds to scan and complete the questionnaire. The survey was conducted entirely in a self-report format. For individuals with reading difficulties, assistance was provided by family members or survey facilitators. Incomplete or abandoned questionnaires were automatically considered invalid. A total of 1200 questionnaires were distributed, and 1062 valid responses were collected, yielding a response rate of 88.5%.

### Ethical Considerations

The study protocol was approved by the research ethics committee of the First People’s Hospital of Yunnan Province (KHLL2023-KY115) and adhered to the principles outlined in the Declaration of Helsinki. Informed consent was obtained electronically from all participants before they began the survey. The electronic informed consent form clearly outlined the study's purpose, procedures, and participants' right to withdraw at any time without consequence. Participants were informed that they could exit the questionnaire at any point, in which case their responses would not be included in the final analysis. If the survey was completed, it was considered as consent to participate in the study. All participant data were anonymized to ensure confidentiality. Participants were not required to provide personal identifiers such as names, and all responses were securely stored. Protective measures were implemented to prevent unauthorized access to the data, and data were processed in compliance with relevant data protection regulations. No compensation was provided to participants for their involvement in the study.

### Analytical Approach to Long COVID and Its Association With Sleep Quality

Comprehensive demographic and medical history data were collected from all participants. Initially, participants were divided into 2 primary groups based on their COVID-19 exposure: the infected group and the noninfected group. Within the infected group, participants were further categorized into the “long COVID group” (those with symptoms lasting more than 4 weeks) and the “non–long COVID group” (those whose symptoms resolved within 4 weeks). To simplify the analysis and enable more direct comparisons between groups, noninfected participants were also included in the “non–long COVID group.” This approach allowed for a clearer contrast between those who experienced long-term symptoms and those who either recovered quickly or were never infected.

Chi-square tests were used to compare variable distributions between groups, with statistical significance set at *P*<.05. To examine the relationship between COVID-19 infection and sleep quality, a 2-tailed *t* test was conducted. Sleep quality metrics, such as subjective sleep quality, were treated as dependent variables, and mean differences between the groups were analyzed. *t* values and *P* values were calculated, with statistical significance defined as *P*<.05.

A linear regression model was then constructed to investigate the relationship between sleep quality, as measured by the PSQI, and various demographic and clinical factors. The PSQI score served as the dependent variable, while demographic and clinical factors were considered independent variables. The regression analysis provided both unstandardized and standardized coefficients for each predictor. *t* values and *P* values were used to assess the relationships between predictors and outcomes, with a significance threshold set at .05. The robustness and validity of the model were further evaluated by reporting detailed metrics.

### Preliminary Analyses for Reliability and Validity Assessment

The reliability analysis revealed standardized coefficients for PHQ-9, GAD-7, PSQI, MSPSS, and AUDIT as 0.924, 0.947, 0.901, 0.977, and 0.889 respectively. Examination of the items revealed that the removal of any single item would not improve these values, indicating no need for item revision or elimination. Overall reliability coefficients for the instruments were strong, ranging from 0.901 to 0.977, reflecting high internal consistency. Additionally, the Kaiser-Meyer-Olkin measure of sampling adequacy for the PHQ-9, GAD-7, PSQI, MSPSS, and AUDIT yielded values of 0.929, 0.943, 0.909, 0.963, and 0.932, respectively, all of which were statistically significant (*P*<.05). These results demonstrate robust validity, confirming the effectiveness of the scales and the reliability of the findings generated by this study (Multimedia Appendix 1).

## Results

### Demographic and Socioeconomic Factors on COVID-19 Outcomes

This study included 1062 participants, with 62.4% (n=663) identifying as male and 37.6% (n=399) as female. Among them, 857 participants were confirmed to have been infected with COVID-19, and 273 developed long COVID. No significant sex differences were observed in infection rates (*P*=.63), though a marginal statistical difference was noted in the prevalence of prolonged symptoms among females (*P*=.051). Age significantly influenced both infection rates and the occurrence of persistent symptoms (*P*<.001 and *P*=.001, respectively), with older age groups being more affected. Specifically, individuals aged 60‐70 years exhibited a higher frequency of both infection and persistent symptoms compared to other age groups. Ethnicity showed a borderline association with infection rates (*P*=.051) but no significant difference in the prevalence of prolonged symptoms. Income did not affect infection rates but was significantly associated with prolonged symptoms (*P*=.01), suggesting that lower income may be linked to longer-lasting symptoms. Higher education levels were correlated with lower infection rates (*P*=.01) and fewer persistent symptoms (*P*<.001). While chronic and mental health conditions did not influence infection rates, they were significantly associated with prolonged symptoms (both *P*<.001). Vaccination status was strongly associated with both lower infection rates and a reduced incidence of long-term symptoms (both *P*<.001; see Table 1).

**Table 1.** Comparative analysis of demographic, socioeconomic, clinical, and psychological factors among COVID-19–infected, noninfected, long COVID, and non–long COVID groups.

Category		Total, n	Infected with COVID-19, n	Not infected with COVID-19, n	*P* value (infection)	Chi-square (*df*)	Long COVID, n	Non–long COVID, n	*P* value (long COVID)	Chi-square (*df*)
**Sex**					.63	0.2 (1)			.051	3.8 (1)
	Male	663	538	125			157	506		
	Female	399	319	80			116	283		
**Age (years)**					<.001	118.1 (3)			.001	16.8 (3)
	<60	39	0	39			39	0		
	60‐70	183	142	41			183	42		
	70‐80	91	81	10			91	30		
	>80	63	49	14			63	14		
**Ethnicity**					.051	3.8 (1)			.46	4.6 (7)
	Han	800	156	644			206	594		
	Zhuang	100	20	80			20	80		
	Yi	81	16	65			25	56		
	Bai	19	1	18			6	13		
	Miao	15	3	12			3	12		
	Hui	14	2	12			5	9		
	Tibetan	12	4	8			1	11		
	Other	21	3	18			7	14		
**Income (renminbi)^a^**					.23	4.3 (3)			.01	12.8 (4)
	>10,000	33	22	11			10	23		
	5000‐10,000	146	119	27			29	117		
	3000‐5000	527	428	99			133	394		
	<3000	356	288	68			101	255		
**Education**					.01	12.8 (4)			<.001	62.8 (4)
	Doctorate or master’s degree	16	15	1			1	15		
	Bachelor’s and college degree	555	444	111			132	423		
	High school diploma and graduate	182	147	35			36	146		
	Middle school and high school	135	121	14			71	64		
	Elementary school and below	174	130	44			33	141		
**Chronic conditions^b^**					.34	10.8 (11)			<.001	81.4 (11)
	None	717	150	567			131	586		
	Diabetes	22	5	17			9	13		
	Benign prostatic hyperplasia	11	3	8			1	10		
	Hyperthyroidism	8	0	8			5	3		
	Hypothyroidism	11	0	11			6	5		
	Rheumatoid arthritis	47	8	39			16	31		
	COPD^c^	56	9	47			22	34		
	Cirrhosis	6	0	6			4	2		
	Malignant tumor	5	0	5			2	3		
	Alzheimer disease	58	10	48			31	27		
	Hypertension	118	19	99			45	73		
	Other	3	1	2			2	1		
**Psychiatric history**					.34	3.4 (3)^d^			<.001	25.7 (3)^d^
	None	884	710	174			201	683		
	Anxiety disorder	98	83	15			42	56		
	Depression	72	56	16			28	44		
	Personality disorder	8	8	0			2	6		
**Vaccination history**					<.001	122.2 (4)^d^			<.001	50.5 (4)^d^
	4 doses	110	46	64			13	97		
	3 doses	754	637	117			188	566		
	2 doses	118	105	13			28	90		
	1 dose	12	12	0			9	3		
	Not vaccinated	68	57	11			35	33		
**PHQ-9** ^e^					.05	7.8 (3)^d^			<.001	48.1 (3)^d^
	No depression	880	701	179			191	689		
	Mild depression	111	96	15			46	65		
	Moderate depression	41	38	3			24	17		
	Severe depression	30	22	8			12	18		
**GAD-7** ^f^					.002	14.6 (3)^d^			<.001	77.3 (3)^d^
	No anxiety	668	516	152			114	554		
	Mild anxiety	243	211	32			89	154		
	Moderate anxiety	80	71	9			33	47		
	Severe anxiety	71	59	12			37	34		
**PSQI** ^g^					.13	5.7 (3)^d^			.049	5.7 (2)^d^
	Very good sleep quality	352	298	54			148	204		
	Decent sleep quality	433	344	89			70	363		
	Average sleep quality	222	172	50			40	182		
	Poor sleep quality	55	43	12			15	40		
**MSPSS** ^h^					.06	5.7 (2)^d^			.01	8.9 (2)^d^
	Low support	146	110	36			31	115		
	Medium support	579	463	116			136	443		
	High support	337	284	53			106	231		
**AUDIT** ^i^					.498	2.4 (3)^d^			.18	5 (3)^d^
	Lower risk	765	612	153			201	564		
	At risk	186	157	29			38	148		
	Harmful	52	40	12			14	38		
	Highly likely dependent	59	48	11			20	39		

a During the study period, the exchange rate was approximately US $1=6.87 renminbi.

b Chronic conditions are ascertained from a list supplied by medical insurance.

c COPD: chronic obstructive pulmonary disease.

d Adjusted value when theoretical frequencies exceed 1 but are less than 5.

e PHQ-9 (Patient Health Questionnaire-9): <10, no depression; 10-14, mild depression; 15-19, moderate depression; ≥20, severe depression.

f GAD-7 (Generalized Anxiety Disorder-7): <5, no anxiety; 5-9, mild anxiety; 10-14, moderate anxiety; ≥15, severe anxiety.

g PSQI (Pittsburgh Sleep Quality Index): <5, very good sleep quality; 6-10, decent sleep quality; 11-15, average sleep quality; 16-21, poor sleep quality.

h MSPSS (Multidimensional Scale of Perceived Social Support): 12-36, low support; 37-60, medium support; 61-84, high support.

i AUDIT (Alcohol Use Disorders Identification Test): 1-7, lower risk; 8-15, at risk; 16-19, harmful; ≥20, highly likely dependent.

### Psychological and Lifestyle Outcomes

In terms of sleep quality, assessed by the PSQI, no significant differences were found between infected and uninfected individuals (*P*=.13). However, the presence of long-term COVID-19 symptoms was significantly associated with poorer sleep quality (*P*=.049). Depression levels, measured by the PHQ-9, were higher across all severity categories in infected individuals compared to the uninfected group, with persistent symptoms further exacerbating depression (*P*<.001). Similarly, anxiety levels, as measured by the GAD-7 scale, were significantly higher in the infected group (*P*=.002), with persistent symptoms also linked to increased anxiety (*P*<.001). Social support, measured using the MSPSS, was marginally lower among infected individuals (*P*=.06), and significantly lower among those with persistent symptoms (*P*=.01). Interestingly, those categorized as having “medium support” reported the highest infection rates and the most persistent symptoms. Regarding alcohol use, assessed by the AUDIT, neither infection status nor the presence of persistent symptoms significantly correlated with alcohol consumption risk (see Table 1).

### Comparative Analysis of Sleep Quality Across COVID-19–Infected and Long COVID Groups

A slight increase in sleep disturbances, subjective sleep quality, use of sleeping medication, and daytime dysfunction was observed in the infected group compared to the uninfected group, although these differences did not reach statistical significance (*P*>.05). However, sleep latency was significantly longer in the infected group (mean 1.73, SD 0.83) compared to the uninfected group (mean 1.57, SD 0.78; *P*= .01). Additionally, total PSQI scores were significantly higher in the infected group (mean 8.52, SD 4.10) compared to the uninfected group (mean 7.76, SD 4.31; *P*=.022). When comparing the non-long COVID group to the long COVID group, the latter exhibited significantly worse outcomes across all sleep metrics, including sleep disturbances, subjective sleep quality, daytime dysfunction, sleep latency, habitual sleep efficiency, sleep duration, and PSQI scores (*P*<.001), with the exception of sleep medication use (*P*=.174) (see Table 2).

**Table 2.** Comparison of sleep quality measures between COVID-19–infected and uninfected groups, and between long COVID and non–long COVID groups.

	Infected with COVID-19^a^ (n=857), mean (SD)	Not infected with COVID-19^b^ (n=205), mean (SD)	*P* value (infection)	Long COVID^c^ (n=273), mean (SD)	Non–long COVID^d^ (n=584), mean (SD)	*P* value
Sleep disturbances	1.20 (0.78)	1.08 (0.81)	.055	1.16 (0.80)	0.93 (0.81)	<.001
Subjective sleep quality	1.19 (0.76)	1.10 (0.84)	.16	1.19 (0.82)	0.94 (0.83)	<.001
Use of sleeping medication	0.49 (0.92)	0.37 (0.83)	.08	0.49 (0.86)	0.33 (0.83)	.17
Daytime dysfunction	0.63 (0.82)	0.57 (0.84)	.30	0.63 (0.87)	0.43 (0.70)	<.001
Sleep latency	1.73 (0.83)	1.57 (0.78)	.01	1.66 (0.80)	1.43 (0.73)	<.001
Habitual sleep efficiency	1.89 (1.07)	1.71 (1.19)	.039	1.87 (1.13)	1.40 (1.21)	<.001
Sleep duration	1.40 (1.10)	1.37 (1.10)	.727	1.47 (1.10)	1.08 (1.04)	<.001
PSQI^e^	8.52 (4.10)	7.76 (4.31)	.02	8.38 (4.09)	6.55 (4.52)	<.001

a Participants who had a confirmed COVID-19 infection.

b Participants who had no evidence of COVID-19 infection.

c Participants whose COVID-19 symptoms lasted more than 4 weeks after the initial infection.

d Participants whose symptoms resolved within 4 weeks, as well as those who were never infected.

e PSQI: Pittsburgh Sleep Quality Index.

### Multivariable Linear Regression Analysis of Self-Reported Sleep Quality in Relation to COVID-19 Infection

Multivariable linear regression analysis was conducted to examine the relationship between COVID-19 infection and subjective sleep quality, as measured by PSQI. The analysis adjusted for demographic factors, prior psychiatric history, previous COVID-19 infection, and emotional well-being. After accounting for potential confounders, no significant relationship was found between COVID-19 infection and overall PSQI scores (*B*=0.104, 95% CI −0.400 to 0.607, *P*=.69).

Nevertheless, several other variables were significantly associated with PSQI scores. Higher education was positively correlated with better sleep quality (*B*=1.828, 95% CI 1.61 to 2.046, *P*<.001). Conversely, lower income and work-related stress were associated with poorer sleep quality (*B*=−1.337, 95% CI −1.654 to −1.021, *P*<.001; *B*=−0.263, 95% CI −0.335 to −0.191, *P*<.001, respectively). Chronic medical conditions and lower levels of perceived social support were also negatively correlated with sleep quality (*B*=−0.083, 95% CI −0.137 to −0.029, *P*=.002; *B*=−0.012, 95% CI −0.022 to −0.001, *P*=.03). Additionally, alcohol consumption was significantly associated with poorer sleep quality, reflected in higher PSQI scores (*B*=0.046, 95% CI 0.020 to 0.072, *P*<.001; see Table 3).

**Table 3.** Factors associated with poor sleep quality—results of linear regression analysis.

Model	Unstandardized regression coefficients		Standardized regression coefficients	*P* value	Regression coefficient 95% CI	
	*B* ^a^	SE	β^b^		Lower bound	Upper bound
Gender	−0.117	0.212	−0.016	.58	−0.534	0.3
Ethnicity	−0.015	0.069	−0.007	.83	−0.15	0.12
Income	−1.337	0.161	−0.276	<.001	−1.654	−1.021
Education	1.828	0.111	0.542	<.001	1.61	2.046
Current domicile	−0.02	0.042	−0.014	.64	−0.103	0.063
Professional role	−0.263	0.037	−0.228	<.001	−0.335	−0.191
Chronic conditions	−0.083	0.027	−0.099	.002	−0.137	−0.029
Psychiatric history	−0.101	0.117	−0.027	.39	−0.332	0.129
Vaccination history	−0.183	0.119	−0.049	.13	−0.418	0.051
COVID infection	0.104	0.256	0.013	.69	−0.4	0.607
Long COVID	−0.924	0.245	−0.125	<.001	−1.404	−0.444
PHQ-9^c^	0.004	0.026	0.006	.89	−0.047	0.055
GAD-7^d^	0.025	0.027	0.04	.36	−0.028	0.078
MSPSS^e^	−0.012	0.005	−0.067	.03	−0.022	−0.001
AUDIT^f^	0.046	0.013	0.106	<.001	0.02	0.072

a *B*: unstandardized regression coefficient.

b β: standardized regression coefficient.

c PHQ-9:Patient Health Questionnaire-9.

d GAD-7:Generalized Anxiety Disorder-7.

e MSPSS:Multidimensional Scale of Perceived Social Support.

f AUDIT:Alcohol Use Disorders Identification Test.

## Discussion

### Principal Findings

According to our findings, there are no significant sex-based disparities in infection rates, suggesting comparable transmission dynamics across male and female populations. However, a slightly elevated incidence of prolonged symptoms was identified in females, a finding that aligns with existing research [24,25]. This phenomenon may potentially be ascribed to underlying factors such as variations in female immune responses, hormonal divergences, or the influence of sex chromosomes [26,27]. Recent studies suggest that these gender-related factors may also play a significant role in the increased risk of long COVID among women, alongside biological differences [28]. A key observation was the strong correlation between advancing age and an increased susceptibility to both COVID-19 infection and the risk of prolonged symptoms. In particular, individuals aged 60‐70 years showed a marked vulnerability. This heightened risk associated with older age may serve as both a predictor and a determinant for the development of long COVID. This trend can be attributed not only to the inherent immunological senescence observed in older populations but also potentially to pre-existing comorbidities or underlying health conditions prevalent within this demographic [29]. While our research underscores the efficacy of vaccination in markedly reducing the incidence of both COVID-19 infection and its prolonged symptoms, the exact connection remains a topic of ongoing debate. Currently, the evidence supporting the idea that vaccination before being exposed to SARS-CoV-2 can reduce the risk of long COVID is limited and not entirely convincing [30]. Further comprehensive studies and empirical substantiation are imperative to corroborate these findings conclusively. The link between education level and factors like infection rates and ongoing symptoms is noteworthy. It suggests that education may play a critical role in raising health awareness, promoting better health practices, and facilitating access to health care resources [31]. Additionally, the connection between income and long-term symptoms indicates that one’s financial situation might influence their access to health care services [32,33]. This brings attention to existing health inequalities, emphasizing the need for policy interventions.

Our research also underscores the multifaceted effects of COVID-19, which extend beyond the immediate physical symptoms of the virus. The findings highlight the complex network of secondary health consequences and the sociopsychological repercussions of the pandemic. Although COVID-19 infection itself does not appear to directly impair sleep quality, lingering symptoms can subtly diminish rest, leading to poorer sleep outcomes. This may be linked to the virus’s interactions with the ACE2 (angiotensin-converting enzyme 2) receptor, which can trigger cellular responses, thrombotic events, and neurological disturbances, contributing to a range of neuropsychiatric symptoms that affect sleep [34]. Individuals experiencing persistent virus symptoms frequently experience isolation and emotional distress, contributing to heightened levels of depression and anxiety [35-37]. These findings are consistent with the impact of the pandemic on overall mental health, further highlighting the long-term negative mental health effects that COVID-19 may have [13,38]. These psychological burdens, combined with ongoing physical discomfort and social isolation, may worsen the mental health outcomes of those affected by long COVID [39].

When compared to the uninfected group, individuals with COVID-19 manifest noticeable alterations in their sleep patterns and perceptions. Although these deviations are evident, they lack statistical significance, underscoring the possibility that the virus may not directly cause severe sleep disruptions. Interestingly, there was not an increased reliance on sleep medications among those infected compared to the uninfected. A deeper examination of objective measures, such as sleep latency, reveals a more nuanced picture. Those infected with the virus exhibited a pronounced delay in transitioning from full wakefulness to sleep, suggesting the virus’s palpable influence on this aspect. While extended sleep latency could be attributed to participants’ memory biases, which might lead them to subjectively report lengthened sleep latency and diminished quality [40], such patterns are genuinely observed in primary insomnia disorders [41]. The significant rise in PSQI scores supports the idea that COVID-19 negatively impacts overall sleep quality. Notably, individuals with long COVID exhibited substantial deficiencies across nearly all sleep metrics when compared to those without prolonged symptoms. This variance is not just a minor deviation but is supported by significant statistical evidence, highlighting the profound and far-reaching effects of long COVID on sleep. These discrepancies likely stem from the impact of inflammation on various aspects of sleep, including latency, duration, and daytime dysfunction [42-44]. Conversely, poor sleep quality can exacerbate the inflammatory response, creating a vicious cycle [45]. While the early stages of COVID-19 may cause only minor sleep disruptions, its lasting effects, particularly in the long COVID population, require close attention and tailored interventions. Given the crucial role of sleep in overall health and well-being, addressing these imbalances is essential for the comprehensive care of COVID-19 survivors.

Considering the systemic and potential neurological impacts of COVID-19, it is reasonable to infer that the virus could directly impair sleep quality. However, through the multivariable linear regression models, we have discerned that this relationship is more complex than initially presumed. After accounting for external factors, the data revealed that socioeconomic, medical, and psychosocial variables play a significant role in shaping sleep quality. Sleep is a multifaceted process influenced by a range of determinants, and our findings indicate that specific socioeconomic, behavioral, and health-related factors can significantly affect sleep quality. Some observations, such as the correlation between higher educational attainment and poorer sleep, warrant further investigation, as they seem to contradict prior studies [46]. It is possible that individuals with advanced degrees may experience more demanding professional responsibilities, which negatively impact their sleep [47]. The negative correlation between income, employment status, and sleep disturbances also invites deeper exploration, possibly reflecting the protective effects of financial stability or the influence of certain job roles in promoting better sleep routines.

Interestingly, our data showed that individuals with chronic conditions reported relatively better sleep quality—a finding that might seem counterintuitive. While initial investigations on COVID-19 revealed limited insights [48], a study highlighted that individuals with chronic conditions showed significant adherence to health guidelines and exhibited a higher inclination to get vaccinated [49]. The structured lifestyles that these patients maintain to manage their conditions might inadvertently benefit their sleep quality. The reinforcing effects of robust social support align well with current research paradigms. It is conceivable that stronger social networks bolster resilience, mitigating feelings of despair which are often linked with sleep disorders [50]. The detrimental impact of alcohol consumption on sleep, while expected, underscores an essential cautionary note. Given that COVID-induced stressors can amplify problematic alcohol consumption, it is imperative to oversee its use, particularly among the infected [51,52]. Future investigative efforts could delve into whether problematic drinking influences the association between COVID-19 and sleep disruptions. Overall, our results provide insight into the complex connections between COVID-19, social factors, health, and sleep. As we face this global health challenge, understanding these links is crucial when helping COVID-19 patients with sleep issues. More research will help us better understand how sleep quality is affected this population.

### Limitations

This cross-sectional study provides insights at a single time point, limiting our ability to make causal conclusions or understand the long-term effects of COVID-19 on sleep. The design restricts the ability to establish temporal relationships and causality between COVID-19 infection, long COVID, and sleep disturbances. Future longitudinal or prospective studies are needed to determine the directionality of these associations and to better inform targeted interventions. Our findings rely on self-reported data, which introduces potential subjectivity and recall bias. This is particularly relevant for participants in the long COVID group, who may experience memory impairments or fatigue that affect the accuracy of their responses. Incorporating objective measures, such as medical records or wearable sleep trackers, in future studies would help strengthen the reliability of the data. Additionally, the recruitment process may have introduced selection bias, as the sample may not fully represent the broader population affected by COVID-19. Participants were recruited via internet, which may have excluded individuals without digital access or those with lower literacy, potentially affecting the generalizability of the findings.

The study exclusively involved Chinese participants (N=1062), which limits the generalizability of the results to other populations and ethnicities. Cultural, genetic, and health care system differences could influence sleep patterns and COVID-19 outcomes. As such, caution should be exercised when extrapolating these findings to global contexts. Future studies would benefit from more geographically and ethnically diverse samples to enhance external validity. Although we controlled for several socioeconomic, health, and psychosocial factors through multivariable regression, some potentially important confounders, such as lifestyle factors (eg, physical activity, diet, and caffeine intake), were not assessed. Including these variables in future research would provide a more refined understanding of the independent contributions of COVID-19 and long COVID to sleep disturbances.

The study used standardized self-report questionnaires to assess sleep quality (PSQI), depression (PHQ-9), and anxiety (GAD-7), which are widely validated but may vary in sensitivity and specificity across different populations and in the context of post-COVID conditions. Moreover, while we identified participants with “persistent symptoms” or “long COVID,” the lack of a clear, consensus-based definition of these terms may introduce some heterogeneity in classification. Future research should adopt standardized diagnostic criteria to ensure consistency and comparability of results across studies. In summary, while this study provides important insights into the relationship between COVID-19, long COVID, and sleep, the above limitations highlight the need for future research to address these methodological challenges and improve the robustness and generalizability of the findings.

### Conclusion

Our comprehensive analysis explores the complex relationship between COVID-19; its prolonged effects, known as long COVID; and sleep quality. We have discovered that while initial COVID-19 infection can lead to minor sleep disruptions, long COVID significantly worsens sleep across various measures. The study further highlights that older adults and those with lower levels of education are more vulnerable to both infection and prolonged symptoms. On a positive note, there is a strong correlation between receiving more COVID-19 vaccine doses and a reduction in both immediate and persistent symptoms. However, it is not just the virus that affects sleep; socioeconomic factors, work conditions, health status, and personal habits also play critical roles in shaping sleep quality for those impacted by COVID-19. Interestingly, higher education levels are associated with poorer sleep, while higher income and stable employment seem to offer protective benefits. Additionally, people with chronic illnesses often report better sleep, potentially due to established self-care routines. Excessive alcohol consumption, however, continues to be a key contributor to poor sleep quality.

In summary, sleep issues related to COVID-19 and its long-term effects are influenced by a range of factors. While the immediate infection may lead to subtle changes in sleep, the lasting symptoms of long COVID can severely impair sleep quality. Comprehensive care for affected individuals should take into account medical, psychological, social, and behavioral factors. Our findings point to specific areas for targeted interventions, particularly for older adults, individuals with lower educational and economic backgrounds, those with limited social support, and those exhibiting harmful behaviors. As COVID-19 continues to evolve, sleep remains a crucial area for ongoing monitoring. The insights from our research provide valuable guidance for patient care and offer a foundation for future studies on post-COVID sleep challenges.

## Supplementary material

10.2196/53522 Multimedia Appendix 1 Questionnaire reliability and validity analysis.

## Abbreviations

ACE2: angiotensin-converting enzyme 2
AUDIT: Alcohol Use Disorders Identification Test
GAD-7: Generalized Anxiety Disorder-7
MSPSS: Multidimensional Scale of Perceived Social Support
PHQ-9: Patient Health Questionnaire-9
PSQI: Pittsburgh Sleep Quality Index
STROBE: Strengthening the Reporting of Observational Studies in Epidemiology
