# Supplementary material for: Investigating the Associations Between COVID-19, Long COVID, and Sleep Disturbances: Cross-Sectional Study
Source: JMIR Public Health Surveill. 2024 Dec 13;10:e53522. doi: 10.2196/53522 (PMC11661692; doi:10.2196/53522)
Supplement: Multimedia Appendix 1 [file publichealth-v10-e53522-s001.docx]

| Questionnaire reliability analysis | | | |
| --- | --- | --- | --- |
| Category | Cronbach α | Cronbach α based on standardized items | Number of items |
| PHQ-9 | 0.924 | 0.925 | 9 |
| GAD-7 | 0.947 | 0.950 | 8 |
| PSQI | 0.901 | 0.907 | 14 |
| MSPSS | 0.977 | 0.977 | 12 |
| AUDIT | 0.889 | 0.901 | 10 |

| Questionnaire validity analysis | | |
| --- | --- | --- |
| Category | KMO (Kaiser-Meyer-Olkin measure) | Bartlett test of sphericity *P* value |
| PHQ-9 | 0.929 | 0.000 |
| GAD-7 | 0.943 | 0.000 |
| PSQI | 0.909 | 0.000 |
| MSPSS | 0.963 | 0.000 |
| AUDIT | 0.932 | 0.000 |

PHQ-9: Patient Health Questionnaire-9.

GAD-7: Generalized Anxiety Disorder-7.

PSQI: Pittsburgh Sleep Quality Index.

MSPSS: Multidimensional Scale of Perceived Social Support.

AUDIT: Alcohol Use Disorders Identification Test.
